# A surface-based morphometry study of risk and resilience markers associated with supramarginal thickness in schizophrenia

**DOI:** 10.1192/j.eurpsy.2021.1094

**Published:** 2021-08-13

**Authors:** A. Tomyshev, M. Omelchenko, V. Kaleda, I. Lebedeva

**Affiliations:** 1 Laboratory Of neuroimaging And Multimodal Analysis, FSBSI Mental Health Research Center, Moscow, Russian Federation; 2 Department Of Youth Psychiatry, FSBSI Mental Health Research Center, Moscow, Russian Federation; 3 Department Of Endogenous Mental Disorders, FSBSI Mental Health Research Center, Moscow, Russian Federation

**Keywords:** schizophrénia, Supragranular thinning, Ultra-high risk of psychosis, Risk and resilience

## Abstract

**Introduction:**

Conventional structural neuroimaging methods can identify changes in cortical thickness but cannot relate these changes to specific cortical layers due to a lack of sensitivity. However, several indirect measures sensitive to changes specifically occurring in supragranular cortical layers were developed recently (github.com/kwagstyl/schizophrenia_gyral_sulcal).

**Objectives:**

The aim was to assess the ability of these novel measures to detect cortical layers thickness characteristics potentially associated with risk or resilience to developing schizophrenia.

**Methods:**

43 first-episode schizophrenia (FES) male patients, 29 non-converted individuals at ultra-high risk of psychosis (ncUHR, mean follow-up period – 6.5 years), and 43 matched healthy controls (HC) underwent structural MRI at 3T Philips scanner. Images were processed via FreeSurfer and MATLAB to derive two markers specific to supragranular thickness change: gyral-sulcal thickness differences (GSTD) and gyral-sulcal intrinsic curvature differences on pial surface (GSCD).

**Results:**

GSCD measures were increased in temporal, parietal and occipital cortices, whereas both GSTD and GSCD were increased in the right frontal cortex in FES compared to HC. No GSTD or GSCD were changed in ncUHR compared to HC, and GSCD was decreased in the frontal cortex compared to FES.

**Conclusions:**

Our findings from the indirect measures indicate a potential predominance of supragranular thinning in FES and suggest that a supragranular thinning in the right frontal lobe might be associated with precipitating risk and/or illness effects of schizophrenia. At the same time, no clear supragranular markers directly associated with resilience or risk mechanisms were identified. The work was supported by RFBR grant 20-013-00748.

